# Comparative Proteomics and Expression Analysis of Five Genes in *Epicauta chinensis* Larvae from the First to Fifth Instar

**DOI:** 10.1371/journal.pone.0089607

**Published:** 2014-02-21

**Authors:** Qiurong Li, Dun Wang, Shumin Lv, Yalin Zhang

**Affiliations:** 1 Key Laboratory of Plant Protection Resources & Pest Management of Ministry of Education, Northwest A & F University, Yangling, Shaanxi, P. R. China; 2 Institute of Entomology, Northwest A & F University, Yangling, Shaanxi, P. R. China; Biogen Idec, United States of America

## Abstract

Blister beetle is an important insect model for both medicinal and pure research. Previous research has mainly focused on its biology and biochemistry, but very little data is yet available in the molecular biology. This study uses differential proteomics technology to analyze the soluble proteins extracted from each of the 5 instars larvae of *Epicauta chinensis.* 42 of the differentially-expressed proteins were identified successfully by MALDI-TOF/TOF-MS. Some of these proteins’ function and their expression profiles are analyzed. Our analysis revealed dynamics regulation of the following proteins: Axin-like protein pry-1 (APR-1), dihydrolipoyl dehydrogenase (DLD), vitellogenin (Vg) and lysozyme C (Lmz-S). APR-1 negatively regulates the Wnt signaling pathway. Its overexpression could result in embryo, leg, eye and ovary ectopica or malformation. DLD catalyzes the pyruvate into acetyl-CoA, the latter is the starting material of juvenile hormone (JH) and ipsdienol biosynthesis through the MVA pathway in insects. While Vg synthesis can be regulated by JH and stimulated by food factors. So DLD may affect the synthesis of JH, ipsdienol and Vg indirectly. The activity of lysozyme is an indicator of the immunity. Nutrition/food should be taken into account for its potential role during the development of larva in the future. Among the five genes and their corresponding proteins’ expression, only *hsc70* gene showed a good correspondence with the protein level. This reflects the fluctuating relationship between mRNA and protein levels.

## Introduction

The blister beetle *Epicauta chinensis* Laporte (Coleoptera: Meloidae) produces the active monoterpene substance called cantharidin. In clinical practice, cantharidin is utilized in curing cancer by restraining the growth of cancer cells. In recent years, cantharidin has also been found to function in killing pests and weeds, as an antiviral and antibiotic, and useful in plant protection [1]–[3]. Cantharidin exists in all developmental stages: egg, larva, pupa and adult. Therefore, the Meloidae are undergoing more research attention.

*Epicauta chinensis* L. occurs with one generation a year in the northern area of China. They are polyphagous insects with adults mainly feeding on fresh soybean and Lucerne (alfalfa) leaves. Larvae feed on locust eggs, such as *Locusta migratoria*, *Oxya chinensis*, etc. or on the larvae and provisions of soil- or wood-nesting bees [4], [5]. This insect undergoes hypermetamorphosis. There are 6 larval instars in *Epicauta chinensis*. The 1^st^ instar is a “triungulin” that is slender, active and well-sclerotized. At this stage they encounter their food source, then excavate and eat the grasshopper eggs. The 2^nd^ to 4^th^ instars are the “first grubs” and are less mobile. These are the primary feeding stages. The 5^th^ instar is a pseudo pupa that does not eat and does not move, but enters diapause. This state can endure about half a year in the natural world; this affects both artificial breeding in the laboratory and our ability to conduct research in a timely manner. The 6^th^ instar, called the “second grub”, is the non-feeding state just prior to pupation [4]–[6]. Such larval development with such marked differences is referred to as hypermetamorphosis, and is perhaps best known in the Meloidae. However, what actually happens within the 5^th^ instar larval body and what factors cause them enter diapause deserves further investigation.

Proteomics is the large-scale study of gene expression at the protein level that provides direct measurement of protein expression levels and insights into the activity of all relevant proteins [7]. Two-dimensional gel electrophoresis (2-DE) separates proteins based on charge and molecular weight. When combined with mass spectrum (MS) and bioinformatics approaches, it provides a powerful tool for understanding complicated organismal processes at the protein level. The techniques have been used successfully in separating insect proteins [8]–[10]. This has expanded research on the fruit fly, mosquito, silkworm, honeybee, and cotton bollworm involving insect immune regulation, physiogenesis, behavior, etc. [11]–[15]. Only a few studies of blister beetles developmental proteomics have become available [16].

This work extends proteomic investigations to larvae growth and development. Comparative analysis of the larval proteome was performed on the 1^st^ to 5^th^ instars. Our main purposes were to (1) identify the most variable proteins within the blister beetle larvae under protein extraction conditions; (2) link the protein variations with major stages of larval growth and development on the basis of their physiological role; and (3) attempt to determine the factors influencing the growth and development of larvae, such as feeding stimulating factors, diapause induction factors, molting factors, and regulating factors for some key metabolic pathways, etc.

## Materials and Methods

### 1. Blister Beetle Larvae

*Epicauta chinensis* L. were raised in our laboratory at Northwest Agriculture & Forestry University, Yangling, Shaanxi, China. Adults were captured in the field in Suide, Shaanxi Province, on July, 2011, and bred indoors. The larvae were reared in plastic cups and maintained under appropriate conditions (30.0±0.5°C, 10.0±1.0% soil humidity, L:D = 16∶8) in incubators. After eclosion, male and female adults were raised in cages. Larvae were reared on *Locusta migratoria* eggs and adults were reared on leaves of *Medicago sativa* (alfalfa).

### 2. Ethics Statement

The blister beetles were not the endangered or protected species in our country and in the world. And the places where *Epicauta chinensis* were captured were all public areas of China. Therefore, no specific permissions were required for our field studies within these public locations.

### 3. Sample Preparation

Larvae for our proteomic and mRNA studies were collected from the soil in cups. Their surfaces were cleaned with 75% alcohol which was then absorbed on filter papers. Samples were grinded to be ultrafines using a mortar and pestle in liquid nitrogen. The majority of this powder was used to extract protein and the remainder was used for RNA extraction.

Powder from the larvae (1 mg larval powder/10 µl buffer) was mixed in Phosphate Buffer Solution (PBS) containing 32.5 mM K_2_HPO_4_, 2.6 mM KH_2_PO_4_, 400 mM NaCl, and a cocktail of protease inhibitors, and placed on ice for 15 min. It was then centrifuged at 12000×g, 4°C for 10 min, and further centrifuged at 15000×g, 4°C for 10 min. The supernatant was removed to another centrifuge tube for further use. The precipitate was mixed with PBS (1 mg larvae/5 µl buffer), then centrifuged at 15000×g, 4°C for 10 min. Supernatant was transferred to another tube and used as a PBS-soluble protein extract. The pellets containing insoluble-proteins were mixed with a lysis buffer (LB) (8 M Urea (SANLAND, Los Angeles, USA), 2 M Thiourea (AMRESCO, Solon, USA), 4% CHAPS (AMRESCO, Solon, USA), 20 mM Tris-base (AMRESCO, Solon, USA), 30 mM DTT (Merck, Darmstadt, Germany), and 2% Bio-lyte (Bio-Rad Hercules, CA, USA), pH 3–10), then homogenized for 2 min on ice and centrifuged at 15000×g, 4°C for 10 min. The supernatant was removed to the tube containing the PBS-soluble proteins extract, and the residual was discarded. Trichloroacetic acid (TCA, Alfa Aesar, Lancaster, USA) was added to the supernatants to reach 10% of the final concentration, and then kept on ice for 10 min for protein precipitation and desalting. This mixture was centrifuged at 15000×g, 4°C for 20 min. Supernatant was discarded and the precipitated protein was washed three times in ice-cold acetone containing 0.2% (wt/vol) DTT, with vigorous disruption by a plastic rod between each wash, and then air-dried. This was then redissolved in the LB containing a cocktail of protease inhibitors (Sigma, Santa Clara, USA) and further ground to help it dissolve. This was brought to room temperature for 2 h, then centrifuged at 15000×g, 4°C for 30 min. The protein concentration was determined according to the method developed by Bradford [17]. This mixture containing the protein extracts of *Epicauta chinensis* larvae was subpackaged and stored at −80°C.

### 4. Two-dimensional Gel Electrophoresis (2-DE)

A total of 300 µg of protein sample was loaded on 17-cm linear IPG strips at pH 5–8 (Bio-Rad Hercules, USA). The final volume of the loading sample was 350 µl. After active rehydration for 16 h, isoelectric focusing (IEF) was carried out at 20°C (Protein IEF Cell, Bio-Rad Hercules, USA) using the following program: step 1: steep and hold for 200 V, 2 h; step 2: gradient 500 V, 2 h; step 3: gradient 1000 V, 4 h; step 4: gradient 8500 V, 5 h; and step 5: steep and hold 8500 V until 70000 Vh. Next, the strips were equilibrated for two intervals of 15 min in an equilibration buffer containing 6 M urea, 50 mM Tris-HCl, 0.07% SDS (AMRESCO, Solon, USA), and 30% glycerol, at pH 7.6. For the first equilibration step, 1% DTT (wt/vol) was added to reduce cystine bridges. Thereafter the proteins were carbamidomethylated with 4% (wt/vol) iodoacetamide. The second dimension was carried out using 12.5% T SDS polyacrylamide gel (1.00 mm). Meanwhile, 10 µl of 2-DE marker was loaded onto a piece of filter paper, and was transferred adjacently to the acid tip of the strip when the filter paper was nearly dry. The protein II Xi Cell (Bio-Rad Hercules, USA) was used to run the second dimension at 20°C in the following steps: 1 W, 45 min; 7 W, 6 h. The gels were then fixed in a solution of 40% methanol and 10% acetic acid for at least 4 h, used Silver Nitrate Staining method and de-stained in water for 12 h prior to scanning.

### 5. Gel Analysis

Gels were scanned by a transparency mode scanner connected to a PC system, at 32-bit red-green-blue colors and 400 dpi resolution for documentation. Images were analyzed using PDQuest ver.7.3.0 (Bio-Rad Hercules, USA). Spots were selected as being differentially-expressed if they showed a >1.5-fold change in spot density and an ANOVA score of <0.05.

### 6. Mass Spectrometry (MS)

For MALDI-TOF/TOF-MS analysis, protein spots were excised and digested [18]. The chosen protein spots were picked manually from the preparative gels using a 1.5-mm picking pen (The Gel Company, San Francisco, USA) and put in Eppendorfs respectively. The excised fragments were washed twice in distilled water, 10 min each time. Ultrasound decoloration used a 1∶1 mix of 50 mM ammonium bicarbonate (NH_4_HCO_3_): acetonitrile (ACN) until the blue faded. ACN was added to make the granule become completely white. This was vacuum-dried for 10 min. Dehydrated gel plugs were bathed in water at 56°C with 10 mM DTT (compounded by 10 µl 1 M DTT, 990 µl 25 mM NH_4_HCO_3_). After cooling to room temperature, this was dried and immersed in 55 mM IAM (55 µl M IAM, 945 µl 25 mM NH_4_HCO_3_) in a dark room for 45 min. The samples were washed three times (25 mM NH_4_HCO_3_ (2×10 min), 25 mM NH_4_HCO_3_+50% ACN (2×10 min), ACN (10 min) and then freeze-dried for 10 min in a vacuum concentrator. The 0.1 µg/µl stock solution of enzyme was diluted 10–20 multiples using 25 mM NH_4_HCO_3_. We then added 2–3 µl to each Eppendorf, centrifuged, and placed it on ice for 30 min. After the solution was absorbed by gel plugs, 25 mM NH_4_HCO_3_ was added to make the total volume 10–15 µl. This was transferred to 37°C for overnight digestion. 1% TFA was added until a final concentration of 0.1% stopped the action; this was shaken to help blending, centrifuged and saved for MS analysis. Each sample (CBB Staining spot, 1 µl; two Silver Staining spots, 3 µl) was applied to a target plate (Applied Biosystems, Foster City, USA) and mixed with 0.1 µl matrix (α-cyano-4-hydroxycinnamic acid in 70% ACN/0.1% TFA) using the dried droplet method [19]. Mass spectrometry was performed using a 4700 MALDI-TOF/TOF Proteomics Analyzer (Applied Biosystems, Foster City, USA). Proteins were identified in the NCBInr database through peptide mass fingerprinting by using MASCOT (Marix Science, London) [20], [21].

All MS data were obtained using the 4000 Explorer software (Version 3.6; Applied Biosystems).

### 7. RNA Extraction and Real-Time PCR

Total RNA was extracted using RNAiso Plus (TaKaRa, USA) and the resulting RNA concentration was measured using Infinite M200 PRO (TECAN, Switzerland). The cDNAs were synthesized individually by reverse transcription using Prime Script RT Reagent Kit (TaKaRa, USA) in 20 µl reaction liquid following the recommended protocol provided by the manufacturer. The gene sequences were obtained from the undisclosed transcriptome data of *Epicauta chinensis*, sequenced by the Institute of Microbiology at the Chinese Academy of Science in Beijing. The sequences of five target genes and one reference gene have been submitted to GenBank and their accession numbers are: KF986611 (*pc*), KF986612 (*hsc70*), KF986613 (*eIF4A*), KF986614 (*eno*), KF986615 (*ugdh*), JQ764814 (*β-actin*). Primer sequences (Table S1) were designed according to their nucleotide sequences using free online primer design tool Primer 3 (http://www.simgene.com/Primer3) or OligoArchitect Online V3.0 (http://www.oligoarchitect.com/Login.jsp). Primers were synthesized by AuGCT (Beijing, China). The PCR reaction was performed on an IQ 5 Optical Module (BioRad, USA). β-actin was used as a reference gene.

### 8. Data Analysis and Processing

Correlation-measured based distances and the UPGMA algorithm were used for the analysis. Protein spot variations were submitted to SPSS 19.0. One-way ANOVAs were used to test the expression differences among five different proteins and their corresponding genes in the five instars larvae. They are presented as mean +/− SEM and differences are considered significant at the P<0.05 level. Figures and tables are produced by GraphPad software.

## Results

### 1. Proteomic Comparison of *Epicauta chinensis* Larvae During Instars 1–5

**Figure 1:**
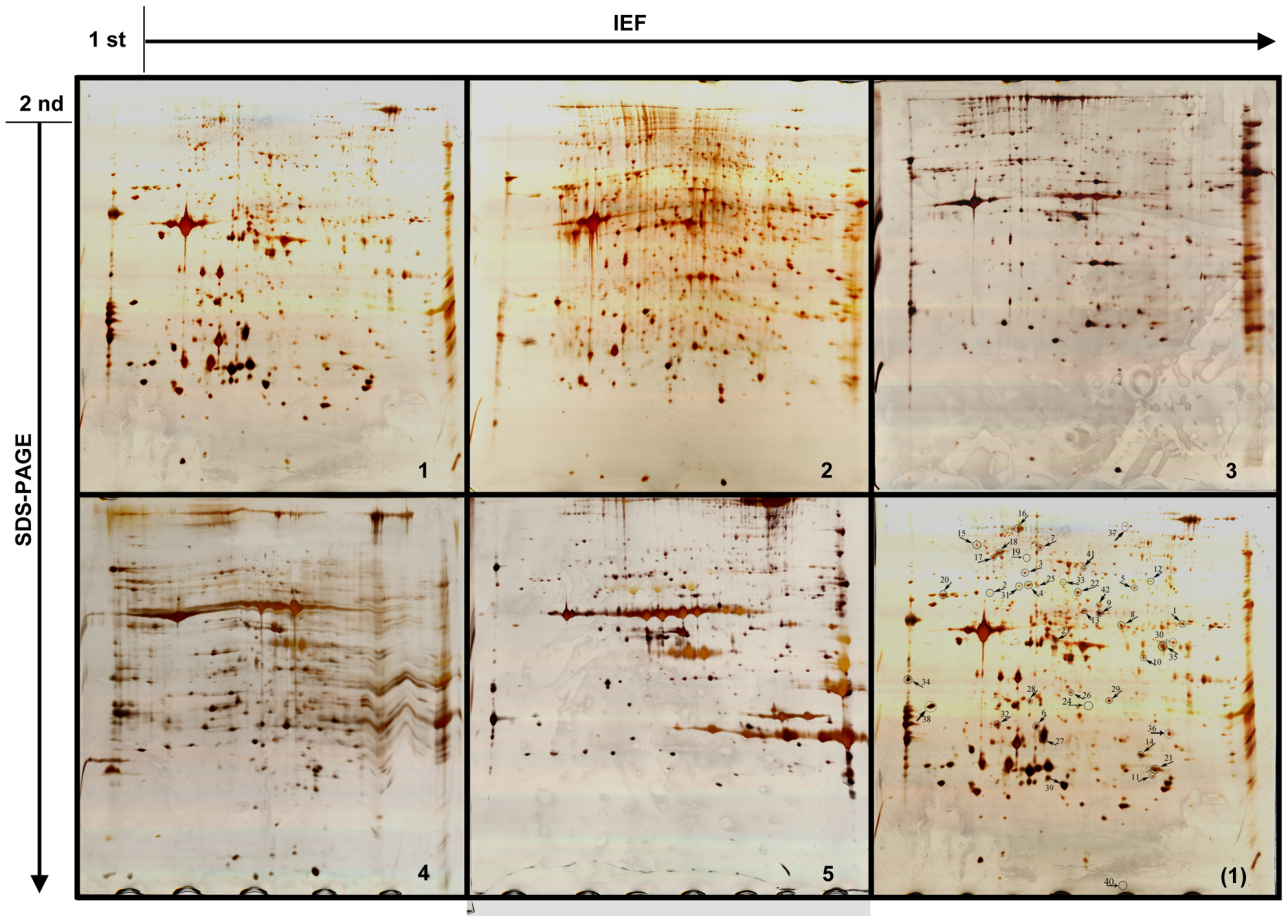
Representative silver-stained 2-DE gels of 1^st^ to 5^th^ instars proteome variation during *Epicauta chinensis* growth. Gels were performed with 300 µg of proteins using 17 cm immobilized pH gradient strips (linear pH5–8) and 12.5% SDS-PAGE was carried out for separation in the second dimension. The differentially-expressed protein spots are from the three replicated gels (n = 3) among 1^st^, 2^nd^, 3^rd^, 4^th^ and 5^th^ instars larvae. They are indicated in gels with a circle, an arrow and an Arabic numeral. The numbers correspond to those in Table 1. The numbers 1, 2, 3, 4, 5 in each gel represents the 1^st^, 2^nd^, 3^rd^, 4^th^ and 5^th^ instar larvae 2-DE gel, and (1) on behalf of the 1^st^ instar 2-DE gel with differential protein spots marked.

Two-dimensional electrophoresis(2-DE) gels of the five different developmental stages were compared and stained with silver nitrate as shown in Figure 1. In the first dimension, the pH range of IPG strips is from 5 to 8 with most of proteins located between pH 6.0 and pH 7.5. Along the second dimension, proteins mainly distributed within the range of 20–70 kDa according to molecular weight (MW). There are prominent differences in body morphology throughout larvae growth from the 1^st^ to 5^th^ instar. The total number of detected spots varied according to the growth stage. In total, about 453 spots, 365 spots, 341 spots, 329 spots, and 314 protein spots were separately detected in the corresponding 1^st^, 2^nd^ 3^rd^, 4^th^, 5^th^ instar larval bodies. On the basis of average intensity ratios of protein spots from 1^st^ to 5^th^ instar, protein spots with a ratio higher than 1.8 or lower than 0.55 were identified as differentially- expressed. 42 proteins were picked and submitted to MALDI-TOF/TOF-MS analysis from these differentially-expressed spots.

### 2. MS Identification of Differentially-expressed Proteins

Forty-two spots were significantly variable (ANOVA) as detected using PDQuest software. They were excised from 2-DE gels and subjected to in-gel trypsin digestion and subsequent MALDI-TOF/TOF-MS identification. The predicted MWs and pHs for a proportion of the identified proteins were generally consistent with the experimental data, as judged from the location of spots on 2-DE gels. However, there were some exceptions. For instance, spots 15, 24, 29, 31, 33, 36, 37, 38, 40 and 42 (Table 1 and Figure 1) had an obvious molecular mass greater than the corresponding identified protein, while spots 2, 4, 5, 19 and 28 had a molecular weight lower than the predicted value. Beyond that, some of the spots (spots 10, 14, 23, 35) distributed in different positions in the same gel were identified as the same protein glycerol-3-phosphate dehydrogenase (GAPDH) or similar to GAPDH which reflects a shift in protein MW or pH. These deviations in molecular weight or pH, may be caused by a variety of factors, including protein degradation, post-translation modification, partial synthesis of proteins during blister beetles larvae development, protein homologues that probably are unique for larvae, or protein translation from selectively spliced mRNAs [22].

**Table 1:** List of proteins identified from the 1^st^ to 5^th^ instar larvae of *Epicauta chinensis.*

Spot no.	Proteinname	Species	Protein ID	Accession no.	Database	Theoretical(pI/Mr)(kDa)	Calculated(2-D)(pI/Mr)(kDa)	Protein score	Sequence Coverage(%)
1	eukaryotic initiation factor 4AII	*Homo sapiens*	gi|485388	BAA06336	NCBInr	5.3/46.6	7.3/42.5	92	23
2	actin homologue	*Ostrinia scapulalis*	gi|315433391	BAJ49799	NCBInr	5.3/30.7	5.7/55.0	78	45
3	GI24228	*Drosophila mojavensis*	gi|195108671	XP_001998916	NCBInr	5.9/57.1	6.0/65.9	47	14
4	PREDICTED: lysozyme C, spleen isozyme-like	*Cavia porcellus*	gi|348580423	XP_003475978	NCBInr	10.6/20.6	6.0/59.2	53	33
5	myosin heavy chain	*Pennahia argentata*	gi|9971579	BAB12571	NCBInr	5.3/22.2	6.9/57.6	74	20
6	PREDICTED: similar to voltage-dependent anion-selective channel isoform 2	*Tribolium castaneum*	gi|91088623	XP_976150	NCBInr	8.6/30.4	6.1/26.6	157	9
7	Heat shock 70 kDa protein cognate 5	*Harpegnathos saltator*	gi|307211659	EFN87680	NCBInr	6.4/75.0	6.1/82.9	239	15
8	PREDICTED: adenosyl homocysteinase	*Macaca mulatta*	gi|297259974	XP_001104495	NCBInr	6.5/49.0	6.8/40.9	212	27
9	Enolase	*Aedes aegypti*	gi|157121051	XP_001653750	NCBInr	6.7/46.9	6.6/45.8	322	22
10	PREDICTED: similar to glycerol-3-phosphate dehydrogenase	*Tribolium castaneum*	gi|91076880	XP_975007	NCBInr	6.8/39.5	7.0/37	141	34
11	Peroxiredoxin 1	*Camponotus floridanus*	gi|307175821	EFN65636	NCBInr	6.3/21.9	7.1/21.1	162	26
**Spot no.**	**Protein** **name**	**Species**	**Protein ID**	**Accession no.**	**Database**	**Theoretical** **(pI/Mr)(kDa)**	**Calculated(2-D)** **(pI/Mr)(kDa)**	**Protein score**	**Sequence coverage** **(%)**
12	UDP-glucose/GDP-mannose dehydrogenase family protein	*Loa loa*	gi|312076607	XP_003140937	NCBInr	6.3/52.9	7.1/61.2	58	22
13	beta-tubulin	*Bombyx mori*	gi|112983318	O17449	NCBInr	4.8/50.6	6.5/46.9	182	48
14	glyceraldhyde-3-phosphate dehydrogenase	*Pyrrhogyra crameri*	gi|269117589	XP_001599507	NCBInr	6.5/24.3	7.0/23.3	115	26
15	PREDICTED:similar to Myosin heavy chain CG17927-PF isoform 5	*Tribolium castaneum*	gi|189239931	XP001813815	NCBInr	5.8/224.7	5.5/84.9	152	15
16	kinesin. putative	*Trypanosoma vivax*	gi|340055502	CCC49821	NCBInr	5.4/110.8	6.0/97.7	49	6
17	NtpA	*Tribolium castaneu*	gi|270006460	EFA02908	NCBInr	4.9/68.4	5.8/70.0	300	14
18	elongation factor 2 (eEF-2)	*Tasmanophilus spinatus*	gi|34597242	AAQ77196	NCBInr	5.8/79.5	5.8/78.9	83	31
19	hypothetical protein TcasGA2_TC010240	*Tribolium castaneum*	gi|270016170	EFA12618	NCBInr	7.2 /27.7	6.0/72.0	96	17
20	PREDICTED: dihydrolipoyl dehydrogenase, mitochondrial-like	*Nasonia vitripennis*	gi|345496470	XP_001602610	NCBInr	8.4/56.5	5.3/55.0	86	22
21	putative actin	*Diaphorina citri*	gi|110456520	ABG74719	NCBInr	5.5/24.7	7.1/21.8	154	62
22	Dihydrolipoyl dehydrogenase, mitochondrial (DLD)	*Camponotus floridanus*	gi|307190023	EFN74243	NCBInr	6.9/54.5	6.5/55.4	119	30
23	glyceraldehyde 3-phosphate dehydrogenase	*Gadus morhua*	gi|25989185	AAL05892	NCBInr	7.7/36.2	6.3/39.4	126	26
**Spot no.**	**Protein** **name**	**Species**	**Protein ID**	**Accession no.**	**Database**	**Theoretical** **(pI/Mr)(kDa)**	**Calculated(2-D)** **(pI/Mr)(kDa)**	**Protein score**	**Sequence coverage** **(%)**
24	PREDICTED: dynein heavy chain 5, axonemal-like	*Meleagris gallopavo*	gi|326917158	XP_003204868	NCBInr	5.9/535.2	6.5/30.4	86	12
25	serum albumin	*Bos indicus*	gi|76445989	ABA42866	NCBInr	6.4/55.5	6.1/61.0	322	37
26	try10	*Macaca mulatta*	gi|58257844	AAW69363	NCBInr	4.6/27.1	6.4/31.5	66	13
27	actin homologue	*Ostrinia scapulalis*	gi|315433391	BAJ4979	NCBInr	5.3/30.7	6.2/25.7	78	45
28	PREDICTED: mitochondrial import receptor subunit TOM22 homolog	*Bombus impatiens*	gi|350411102	XP_003489240	NCBInr	4.0/16.1	6.1/30.1	57	54
29	PREDICTED: cytoplasmic dynein 1 heavy chain 1	*Oryctolagus cuniculus*	gi|291410977	XP_002721753	NCBInr	6.0/530	6.7/30.2	101	11
30	hypothetical protein TcasGA2_TC014998	*Tribolium castaneum*	gi|270008484	EFA04932	NCBInr	7.8/40.1	7.2/39.3	71	11
31	serum albumin precursor	*Bos taurus*	gi|30794280	NP_851335	NCBInr	6.1/71.3	6.0/58.3	79	23
32	splicing factor 3a, subunit 2, isoform CRA_c	*Mus musculus*	gi|148699551	EDL31498	NCBInr	9.5/22.2	5.8/26.9	80	47
33	hypothetical protein	*Homo sapiens*	gi|57999440	CAI45931	NCBInr	6.2/232.3	6.3/58.0	72	15
34	PREDICTED: tropomyosin-1-like isoform 1	*Bombus terrestris*	gi|340723822	XP_003400287	NCBInr	4.5/32.2	5.1/33.4	300	24
35	PREDICTED: similar to glycerol-3-phosphate dehydrogenase	*Tribolium castaneum*	gi|91076880	XP_975007	NCBInr	6.8/39.5	7.1/39.0	285	32
**Spot no.**	**Protein** **name**	**Species**	**Protein ID**	**Accession no.**	**Database**	**Theoretical** **(pI/Mr)(kDa)**	**Calculated(2-D)** **(pI/Mr)(kDa)**	**Protein score**	**Sequence coverage** **(%)**
36	unnamed protein product	*Oikopleura dioica*	gi|313228938	CBY18090	NCBInr	5.7/70.5	7.2/25.8	62	19
37	PREDICTED: similar to carboxylase: pyruvate/acetyl-coa/propionyl-coa	*Tribolium castaneum*	gi|91080283	XP_973877	NCBInr	6.9/253.4	6.8/99.2	206	38
38	Protein Y71G12B.11, isoform a	*Caenorhabditis elegans*	gi|25143518	NP_490886	NCBInr	5.8/280.7	5.2/29.4	84	19
39	stat3	*Bubalus bubalis*	gi|108744047	ABG02396	NCBInr	6.3/20.2	6.3/20.2	45	29
40	focal adhesion kinase	*Drosophila prostipennis*	gi|63147881	AAY34266	NCBInr	8.3/35.4	6.9/12.0	76	39
41	Axin-like protein pry-1	*Ascaris suum*	gi|324508120	ADY43431	NCBInr	8.8/66.0	6.5/68.6	45	22
42	vitellogenin	*Spodoptera litura*	gi|156481320	ABU68426	NCBInr	9.0/199.2	6.6/49.3	68	15

Data obtained for these spots are presented in Table 1 and spot positions are illustrated on the gels in Figure 1.

### 3. Bioinformatics Analysis of Differentially-Expressed Spots by Functional Classes

**Figure 2:**
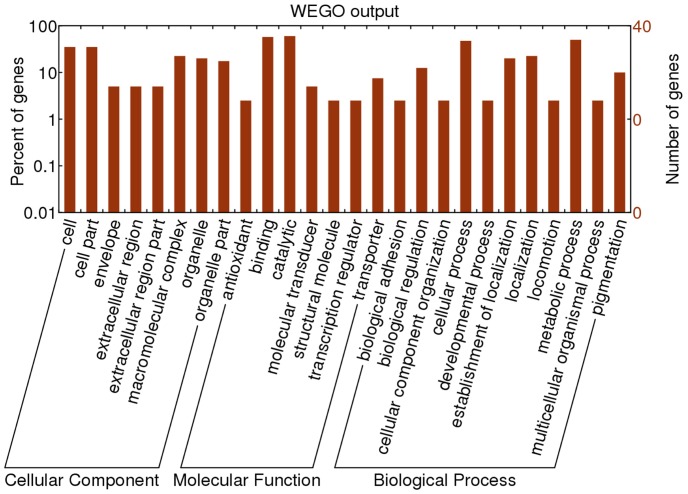
Assignment of the identified spots into putative functional categories. GO categories of the differentially-expressed proteins from the 1^st^ to 5^th^ instars of *Epicauta chinensis* larvae by WEGO online software. The number of genes shows the amount of genes with available GO terms. The percent of genes shows the proportion of total genes. These proteins were classified into 3 main categories and 26 subcategories.

The identified proteins were submitted to http://wego.genomics.org.cn/cgi-bin/wego/index.pl for GO (gene ontology) annotation. The 42 proteins were classified into three groups as shown in Figure 2, including cellular components, molecular function and biological process. At the level of cellular component, most of the differentially-expressed proteins were involved in cells and cell parts. Among biological processes, cellular and metabolic processes comprised the largest proportion. Molecular functions include antioxidant, binding, catalytic, molecular transducer, structural molecule, transcription regulator and transporter. The highest proportion of proteins fell into binding and catalytic functions. A few of them showed stage specificity, for instance, some proteins expressed mostly in the 4^th^ instar mainly participated in metabolic processes while some proteins that reached their highest expression level in the 5^th^ instar had a catalytic activity.

### 4. Dynamics of Protein Networks during 1^st^ and 5^th^ Instar

**Figure 3:**
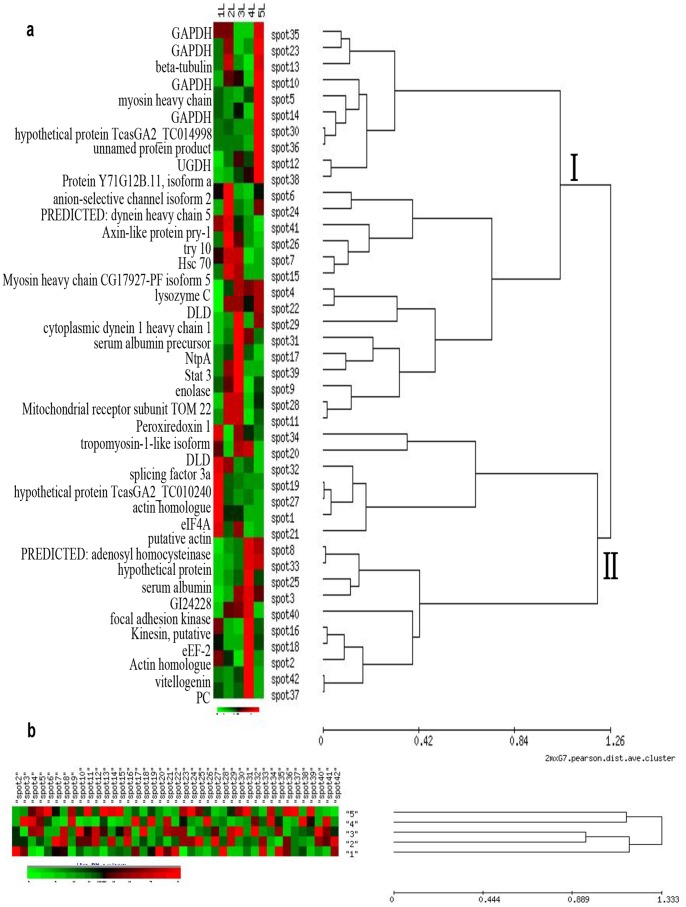
Hierarchical clustering analysis of the 42 identified spots. (**a**) spots listed in Table 1 were clustered according to their percentage of volume from 1^st^ to 5^th^ instar by the method of UPGMA. The spot number is indicated on the right of the heat plot, the protein name is on the left side. Two main clusters were formed. (**b**) Cluster according to the stages of larvae growth. Numbers “1”, “2”, “3”, “4”, “5” are used instead of the 1^st^, 2^nd^, 3^rd^, 4^th^ and 5^th^ instar. The five instars were clustered into three groups.

To summarize the information contained in Table 1 and to cluster the proteins showing similar expression profiles during the larval growth and development, hierarchical clustering was applied to the 42 identified spots (Figure 3a). Spots were classified according to their pattern of volume variation from the 1^st^ to 5^th^ instar using the unweighted pair group method with the arithmetic method (UPGMA). The 42 spots were clustered into two groups. Cluster I was composed of 25 proteins whose overall abundance increased during larval growth. The first subcluster (from spots 35–38) comprised 10 spots. Among them, there were 8 spots (from 35 to 36) whose abundance increased from the 1^st^ to 2^nd^ instar, decreased in the 3^rd^ and 4^th^ and then increased dramatically in the 5^th^. The other two spots (spot 12 and 38) had a protein expression index that kept increasing and reached the highest level in the 5^th^ instar. A second subcluster (from spots 6–15) comprised 6 spots whose abundance increased in the 2^nd^ rather than 1^st^ instar, then decreased in the 3^rd^, and declined to the lowest abundance in the 5^th^ instar. The cluster I also included 9 spots (from 4 to 11) whose trend of protein expression increased from the 1^st^ to 3^rd^ instar, decreased at the 4^th^ but increased again at the 5^th^ instar, and had the largest expression amount at the 3^rd^ instar.

Cluster II was comprised of 17 spots. Seven (spots 34–21) showed a higher level of abundance in the 1^st^ instar, whereas the rest of the ten protein spots (spots 8–37) reached their maximal abundance in the 4^th^ instar. Among these spots, it is worth noting that the important material of egg and larva growth - vitellogenin (spot 42, Vg), it was considerably expressed in the first, second and third instars, reaching a peak at the fourth instar, then decreased in the fifth instar.

As shown by the transposed cluster tree (Figure 3b), the age effect is the primary factor explaining spot variations. The five stages were clustered into three sub clusters which corresponded to 1^st^ instar (three-jaw type), 2^nd^–3^rd^ instars (melolonthoid) and 4^th^–5^th^ instars (melolonthoid and pseudo pupa).

### 5. Translation and Transcription Level Expression

**Figure 4:**
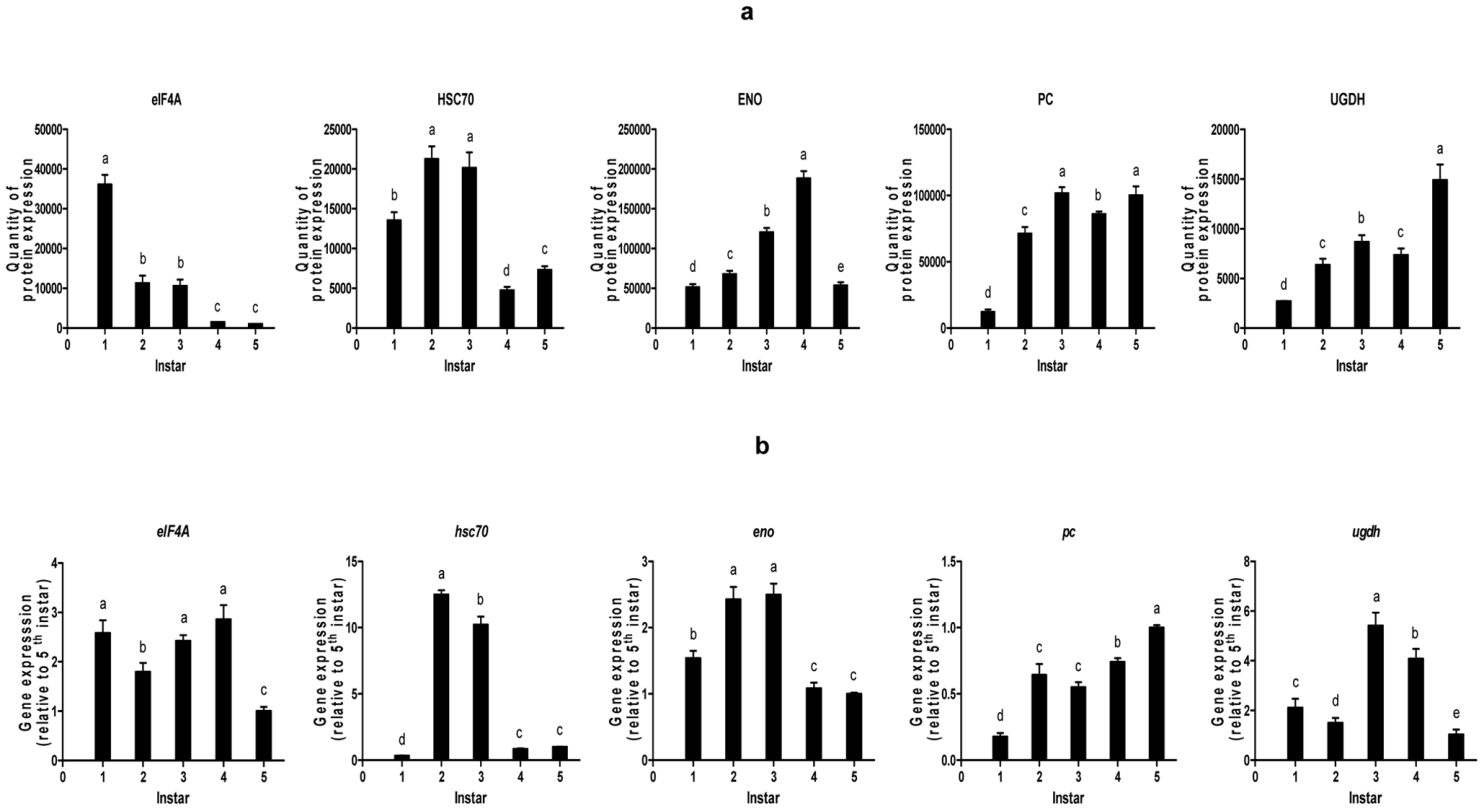
Expression pattern at protein and mRNA level. (**a**) Differential expression profiles of the 5 identified proteins. The plots show the mean standardized log abundance derived from the three replicated gels (n = 3) among 1^st^, 2^nd^, 3^rd^, 4^th^ and 5^th^ instars larvae. Different letters indicate when the expression quantities were significantly different. Master spot number and abbreviation of the proteins’ names are indicated on top of each graph. (**b**) Gene expression patterns. Gene expression of candidate genes in blister beetles larvae of 1^st^, 2^nd^, 3^rd^, 4^th^ and 5^th^ instars, respectively. Expression levels are given relative to the 5^th^ instar. Significant differences are indicated by different letters. ** The numbers 1, 2, 3, 4, 5 represent the 1^st^, 2^nd^, 3^rd^, 4^th^ and 5^th^ instar respectively.

To assess the correspondence between protein expressional abundance and transcriptional activity, 5 genes were selected for RT-PCR analysis (Figure 4b). The data showed that only one gene *hsc70* was in full accord with what registered at protein level (Figure 4a). This extent of change was very significant, a change of up to 37-fold in *hsc70* mRNA level only resulted in a 1.6- fold increase in hsc70 protein level in the 2^nd^ over 1^st^ instar. In some cases, the relationship between mRNA abundance and protein functionality is relatively straightforward, with apparent up-regulation of pc (propionyl-CoA carboxylase) and *pc* gene both in the 2^nd^ and 5^th^ instars. But there might be a discrepancy in, e.g. eIF4A (eukaryotic initiation factor 4AII), its expression in the mRNA level kept nearly in 1^st^, 3^rd^ and 4^th^ instars, while it down-regulated distinctly in the corresponding stages at the protein level; *enolase* (*eno*) down-regulated in the 4^th^ instar at mRNA level yet enolase maintained a continuous increase from 1^st^ to 4^th^ instars at protein level. In addition, *ugdh* gene down-regulated in the 5^th^ instar whereas its protein up-regulated obviously.

## Discussion

### 1. Function Analysis of Some Differentially-expressed Proteins

In this study, we employed comparative proteomics and successfully identified 42 spots from the blister beetle larvae by using MALDI-TOF/TOF-MS. Most of them were found to be related with the processes of substance and energy metabolism, nutrient digestion and absorption, and innate immunity.

#### Proteins Relevant to Substance and Energy Metabolism

We found thirteen proteins were related to the metabolism of carbohydrate and energy production involved in glycolysis (spots 9, 10, 14, 23, 30, and 35), synthesis of fatty acids (spot 37), metabolic processes of fructose and mannose (spot 12), the synthesis of hub substances for energy and substance metabolism and processes of glycolysis and the citric acid cycle (spot 22), and ATP generation (spots 5, 8, 16 and 40).

GAPDH transforms 3-glyceraldehyde phosphate into 1,3-diphosphoglycerate in the glycolysis process. It acts as reversible metabolic switch under oxidative stress [23]. In addition, it has other physiological functions, such as initiating apoptosis [24], membrane fusion, vesicle trafficking, a chaperone of phosphotransferase, DNA repairing and transcriptional regulation [25]. Enolase is known to be a multifunctional protein, e.g. as a glycolytic enzyme, plasminogen-binding protein and heat-shock protein [26], [27], participates in the regulation and control of transcription, apoptosis and cell differentiation [28]–[30]. Enolase functions as a virulence agent in early egg establishment at *Aphidius ervi* oviposition in aphid hosts [31]. Its expression level increased gradually from 1^st^ to 4^th^ instar, then decreased back to near the level of the 1^st^ instar when they were the 5^th^ instar larvae. This may be associated with nutrition because the 1^st^ and 5^th^ instar larvae do not feed, while the 2^nd^, 3^rd^ and 4^th^ instars are the major feeding stages.

Dihydrolipoyl dehydrogenase (DLD), also known as dihydrolipoamide dehydrogenase is the compound enzyme which catalyzes pyruvate into acetyl-CoA. It is a type of pivotal carbohydrate during energy metabolism, e.g. the citric acid (TCA) cycle and mevalonic acid (MVA) pathway. The Juvenile hormones (JHs) which are a group of acyclic sesquiterpenoids can regulate development, reproduction, diapause, and polyphenisms of insect [32], [33]. Its biosynthetic pathway in insects is divided into two main components: the early steps, acetyl-CoA up to farnesyl diphosphate (FPP), belong to the MVA pathway while the later steps effect the conversion of FPP into JH [34]. Besides, the male pine engraver *Ips pini* contains one type of monoterpenoid pheromone called ipsdienol. It is synthesized in the males’ midgut by the MVA pathway [35]. As the starting material of the MVA pathway, acetyl-CoA is first catalyzed to generate the C5 isopentenyl diphosphate (IPP), which then undergoes two successive condensations to generate FPP [36]. In the case of enough precursor pyruvate, the activity of the catalyzing enzyme DLD determines acetyl-CoA’s production, thus influences the content of JH and ipsdienol indirectly.

#### Differentially-expressed Proteins Involved in Digestion and Absorption of Nutrients

In this study, four proteins which were identified as Myosin heavy chain CG17927-PF isoform 5 (spot 15), axonemal-like (spot 24), Cytoplasmic Dynein 1 heavy chain 1 (spot 29) and Tropomyosin-1-like isoform 1 (spot 34) may function in digestion and absorption of nutrients (Figure 1 and Table 1). These proteins are related to the actin filament through driving myosin molecules to move along the actin filaments [37], stabilizing actin filaments [38] and inhibiting actin filament formation [39]. We found that the Myosin heavy chain CG17927-PF isoform 5 and Dynein Heavy chain 5 had the highest expression in the 2^nd^ instar and then decreased gradually at later stages. Cytoplasmic Dynein 1 heavy chain 1 expressed most in the 3^rd^ instar. The decreased expression of the Myosin heavy chain causes the destabilization of the apical brush border membrane; this will result in increased sensitivity to oral infection by bacterial pathogens [40]. While Tropomyosin-1-like isoform 1 expressed the highest amount in the first instar, it then decreased gradually. The decreased expression of Tropomyosin-1-like isoform 1 inhibits the formation of the actin filament and thereby weakens the contraction ability of the smooth muscles in the blister beetle midgut.

#### Proteins Related to Blister Beetle Larval Innate Immune System

Hsc70 apparently down-regulated in the fourth and fifth instars. It is in the HSP family. HSPs are produced as a protective response by cells to a variety of stress factors. The major physiological functions of the Hsp70 superfamily’s members are protein folding, unfolding and translocation [41]. Hsp70s are the most conservative and important subfamily and they are the most sensitive in responding to stress. They could protect cells from stimulus and injury as well as promote the repair of damaged cells as well as have anti-inflammatory and anti-apoptotic effects. They also provide attractive targets for immune responses towards pathogens [42], [43]. Expression levels of Hsc70 are positively correlated to the level of tissue cells resistance to damage and their ability to protect themselves.

Vitellogenin (Vg) is the key component of insect eggs and is the main source of nutrition for early development of eggs and larvae. It has the function of carrying and transporting fat as well as having immunological properties [44]. Vg is produced in the fat body of insects [45], [46]. Its synthesis could be stimulated by food factors [47]. For some species of the investigated insects, e.g. *Drosophila melanogaster, Locusta migratoria*, it has been documented that JH stimulates the transcription of the vitellogenin genes and the consequent control of vitellogenin production [48], [49]. However, in some other insects such as *Helicoverpa zea*, *Lymantria dispar*, honeybee and so on, JH is proposed to act as a repressor of Vg synthesis [50]–[52]. A low JH titre in honey bee workers permits the onset accumulation of Vg in haemolymph, whereas high JH levels turn off Vg synthesis [53]. The *Drosophila melanogaster* and *Locusta migratoria* are attached to Diptera and Orthoptera respectively. While *Helicoverpa zea* and *Lymantria dispar* are members of Lepidoptera and honeybee belongs to Hymenoptera. The promotion and repression impact on Vg brought by JH may attribute to the different biosynthesis process of Vg and JH in different insects, or different insects’ diverse physiological processes or functions.

Lysozyme C (Lmz-S) is a type of alkaline enzyme which can hydrolyze mucoitin in pathogenic bacteria. It acts on the extraneous antigen that then makes it dissolve or decompose. This enzyme has anti-bacterial, anti-inflammatory and anti-viral activities and is also a cold adapted protein [54], so it has a role in defense and immunity. Lmz-S exhibites considerable catalytic activity at low temperatures and low activation energies [55]. Lysozyme activity can be detected by the resonance scattering spectra of the micrococcus method, lysodeikticus turbidimetric and colorimetric methods, etc. to assay the level of immunity [56]. The activity of Lysozyme develops in haemolymph of immature stages of *Bomyx mori* and *Galleria mellonella* after injection with microorganisms but not after saline injection [57].

It has been reported that dietary influences the immunity of an organism [58]. The immunity is stronger after feeding compared with those have not fed [59]. And a dietary with supplementation improved the immunity of *Lumbricus terrestris* [60]. So we conjecture that this may be relevant with blister beetles’ feeding. The sampled 1^st^ instar larvae of blister beetles have not fed, so those immune system proteins’ levels are the lowest and their immunity is the lowest. The second and third instars have a longer feeding period thus promoting the expression levels significantly. The fourth stage larvae eat less, while the fifth stage larvae do not feed at all. Therefore most are down-regulated in the 4^th^ and 5^th^ instars to some extent, but still express higher than in the 1^st^ instar.

#### Proteins Related to Egg Development and Adult Limb Formation

Axin-like protein pry-1 (APR-1) is a typical negative regulator of the Wnt/wingless signaling pathway and it regulates the stability of the Wnt/wingless pathway effector beta-catenin [61]. *Tribolium castaneum* female adults injected with *Tc-axin* dsRNA produced progeny phenotypes that ranged from mildly affected embryos with cuticles displaying a graded loss of anterior structures, to defective embryos that condensed at the posterior pole in the absence of serosa [62]. Ectopic expression of *axin* induces notches in the wing, generation of a supernumerary leg from the ventral side of the normal leg and loss of the sternite structure in the abdomen. Furthermore, overexpression of *axin* results in eye and ovary ectopica in *Drosophila melanogaster* [63].

### 2. Gene-Nutrient/Food Interaction

Many genes’ expression depends on the environment in which they survive and involves the food they take in. A nutrient is regarded as a vital environmental factor and there are extensive interactions between nutrients and genes [64], [65]. In this study, we found that as one type of nutrient formed, diet could alter the expression of proteins related to carbohydrates and energy metabolism, immunity, digestion and absorption of nutrients. For example, lysozyme C, adenosyl homocysteinase, dihydrolipoyl dehydrogenase, mitochondrial import receptor subunit TOM 22 homolog, hypothetical protein, and vitellogenin may be affected by nutrient factors. Mitochondrial import receptor subunit TOM 22 homolog is the outer mitochondrial membrane transporter enzyme. Its function is to help the protein across the mitochondrial outer membrane, mediate the signal peptide of mitochondrial protein into the cavity between the mitochondrial membrane and help the proteins which have penetrated insert into the mitochondrial outer membrane. This protein spot has a lower expression in the 1^st^ instar, but it accelerates suddenly in the 2^nd^ instar after feeding. We infer that the activity of this outer mitochondrial membrane transporter enzyme is possibly connected with stimulation from food factors. As a consequence, nutrients affect growth and development as well as the resistance to pathogens in blister beetles.

### 3. Comparation at Protein and mRNA Level

According to comparative results of the five proteins and corresponding five genes expression profiles, we found there were discrepancies between the protein and mRNA levels. It may be attributed to the following reasons. First, the process of mRNA translating to proteins is downstream of gene expression and this process may generate variability e.g. post-transcriptional modification and regulation [66]. Second, when gene expression down-regulates but protein expression up-regulates, the gene has not been positively-regulated at the mRNA level, and there is a constitutively-expressed protein regulated by an intracellular activator that becomes the active form. Beyond that, the instability of mRNA is an important source of randomness in gene expression, especially when mRNA levels are low. Therefore a direct relationship between protein and mRNA levels cannot always be assumed or expected [67], [68].

### 4. Cantharidin Biosynthesis Pathway

The same as JH and ipsdienol, cantharidin is also a kind of terpenoid. There are two pathways: the MVA pathway and the non-MVA pathway in which terpenoid biosynthesis in organism have been discovered by scientists. So far, the non-MVA pathway is mainly found in plants, protists and microorganisms [69]. While the MVA pathway exists in many viruses and in all higher eukaryotes including insects [35], [70]. Researchers have not found the non-MVA pathway in animals up to now.

Because of cantharidin consists of 10 C atoms, at first it was thought synthesized by acetate through the polyketide (PK) pathway or MVA pathway, with the latter more likely. However, Guenther *et al.* [71] verified that cantharidin could not be synthesized by acetate through degradation experiment. McCormick, *et al.* [72] have demonstrated that cantharidin is produced by degradation of the farnesol carbon skeleton and suggested the possibility of cantharidin as a juvenile hormone metabolite by tracing the source of oxygen atoms in cantharidin using the isotope-labeling method. Peter *et al* [73] also discovered farnesol takes part in cantharidin biosynthesis in beetles. Schlatter *et al.* [74] found all of the H atoms in cantharidin come from farnesol directly and derive from mevolate except for the H atom of C_6_. The process, from acetyl-CoA to farnesyl diphosphate (FPP), belongs to the MVA pathway. A soluble form of phosphatase is capable of converting FPP into farnesol [75].

Though some kinds of terpenoid are synthesized by MVA pathway, however, due to they have different chemical construction and are attached to different orders and families of insects, so whether cantharidin is indeed biosynthesized by the MVA pathway in blister beetles, there is no definite evidence that can prove it so far.

## Conclusion

In this work, we employed a comparative proteomic approach to investigate the proteomic differences of *Epicauta chinensis* larvae from first to fifth instar. 2-DE profiles showed variations in some spots’ abundance. 42 differentially-expressed proteins were identified successfully by MALDI-TOF/TOF. The identified differentially-expressed proteins Vg, DLD and lysozyme C take important roles in blister beetle larvae development. Vg supplies nutrients for eggs and larvae growth and its biosynthesis can be regulated by JH and stimulated by food factors. DLD catalyzes the pyruvate into acetyl-CoA then further affects biosynthesis of JH, ipsdienol and Vg. Lysozyme activity is an indicator of the immunity. GO function and cluster correlation analysis results showed the differential proteins’ expression had stage characteristics. The identified proteins expressing higher in the 1^st^ and 5^th^ instars mainly participate in the metabolism of basic carbohydrates. The first and fifth instars are two stages which larvae do not feed. While the 2^nd^, 3^rd^ and 4^th^ instars are the active feeding stages, proteins responsible for their immune system, the nervous system and regulation of cell growth and development are active. We suggest that the proteins’ expression is related to the stimulation of food to some extent. In this study, only the heat shock family protein (hsc70) shows a good correspondence. There is no direct relationship between protein and gene expression. In general, further studies such as genomic function identification, systematic biology-based research and additional extensive proteomic studies are necessary to advance research in this area.

## Supporting Information

Table S1 The representative gene’s name and its RT-PCR primer.
(XLSX)
